# Drug-target binding affinity prediction using message passing neural network and self supervised learning

**DOI:** 10.1186/s12864-023-09664-z

**Published:** 2023-09-20

**Authors:** Leiming Xia, Lei Xu, Shourun Pan, Dongjiang Niu, Beiyi Zhang, Zhen Li

**Affiliations:** College of Computer Science and Technology, Qingdao University, Qingdao, China

**Keywords:** Drug-target binding affinity, Self-supervised learning method, Molecular representation, Protein representation

## Abstract

**Background:**

Drug-target binding affinity (DTA) prediction is important for the rapid development of drug discovery. Compared to traditional methods, deep learning methods provide a new way for DTA prediction to achieve good performance without much knowledge of the biochemical background. However, there are still room for improvement in DTA prediction: (1) only focusing on the information of the atom leads to an incomplete representation of the molecular graph; (2) the self-supervised learning method could be introduced for protein representation.

**Results:**

In this paper, a DTA prediction model using the deep learning method is proposed, which uses an undirected-CMPNN for molecular embedding and combines CPCProt and MLM models for protein embedding. An attention mechanism is introduced to discover the important part of the protein sequence. The proposed method is evaluated on the datasets Ki and Davis, and the model outperformed other deep learning methods.

**Conclusions:**

The proposed model improves the performance of the DTA prediction, which provides a novel strategy for deep learning-based virtual screening methods.

## Background

The drug-target affinity is of great importance for drug discovery and screening. Generally, it can be obtained through biological experiments. However, the time costs and economic costs of the experiment are huge. Therefore, researchers try to use the powerful computational abilities of computers to alleviate the difficulties in drug discovery. In this paper, we focus on how to utilize computer technology to predict the drug-target binding affinity.

In recent years, deep learning plays an essential role in computer and other fields. Hinton et al. [1] first introduced the concept of deep learning (DL), with the advancement of DL and the growth of drug-related data, many DL-based methods were applied in various steps of drug discovery [2, 3]. For the drug-target binding affinity (DTA) prediction, DL was used to learn and understand information from both molecules and proteins to determine whether a pair of drug and target could be bound together (classification tasks) or to predict the DTA value (regression tasks).

In the DTA prediction task, the first challenge is how to select the appropriate representation of the experimental data. A common molecular representation method is the Simplified Molecular Input Line Entry System (SMILES) [4], and proteins can be represented as amino acid sequences. The recurrent neural network (RNN) is an effective method for extracting protein and molecular features from sequential data. Zheng et al. [5] extracted potential semantic information between protein and molecule through Long Short-Term Memory Network (LSTM), a special recurrent neural network, for drug-target interaction (DTI) prediction. DeepH-DTA [6] used a bidirectional ConvLSTM [7] to model spatial sequence information on SMILES data. GLSTM-DTA [8] combined the graph neural network (GNN) and the LSTM for molecule and protein representation in DTA prediction, respectively. However, the RNN only considers the context of sequences without the original structure of molecules, which will affect the generalization ability of the model on other datasets.

For the molecule, the complete structural information of molecules is contained in the 3D atomic coordinates, but their 3D grid representation has a large number of redundant voxels where no atoms exist, resulting in inefficient computations. In addition, the rotationally invariance and scale invariance are both need to be solved in the 3D grid, which could affect the prediction of the binding affinity. In contrast, the 2D graph representation is compact and rotation invariant, which ensures the stability and repeatability of model predictions. The molecule could be converted to the graph, where atoms and bonds are represented as nodes and edges in the graph. GraphDTA [9] constructs graphs to describe molecules and apply GNN for feature extraction in DTA prediction. MGraphDTA [10] also proposed a multiscale graph neural network for DTA prediction. Moreover, graph convolutional neural networks (GCN) could be used for molecular representation [11]. For example, Ying et al. [12] combined efficient random walk and graph convolution networks to generate atom embeddings that contain information of graph structure and atom. WGNNDTA [13] proposed a weighted graph neural network to provide more detailed information on the residue interaction for DTA prediction. GanDTI [14] designed a Residual Graph Neural Network to extract the embedding of molecules for DTA and DTI prediction. Shao et al. [15] used GCN to extract features from the drugs and targets, respectively, and the convolutional neural network (CNN) was used to extract and predict potential associations between drugs and targets. For the graph representation, the major challenge is how to update messages between atoms effectively. Message Passing Neural Network (MPNN) [16] was proposed for molecular property prediction, which updated the atom information in the molecular graph while ignoring the information from the bond. To alleviate this problem, CMPNN [17] improved the molecular embedding method considering that the information from chemical bonds to atoms is equally essential.

For the protein, 3D protein representations could be used in DTA prediction to obtain more precise results. However, there are relatively few protein data with known 3D structures, and the use of large amounts of 3D information may lead to high sparsity problems, which could affect the performance of the model. On the other hand, the one-dimensional amino acid sequences are easily acquired and could be naturally processed by models in the field of natural language processing(NLP) for representation.

The Bidirectional Encoder Representations from Transformers (BERT) [18] used the mask language model (MLM) to mask some input tokens for prediction to learn the accurate representation of the words. Also, the BERT was applied in the bioinformatics field. Ho et al. [19] extracted and analyzed contextual word embeddings from a pre-trained BERT model to explore similarities in natural language and protein sequences for flavin adenine dinucleotide binding sites prediction. MOLBERT [20] used the transformer architecture of BERT to learn the representation of molecules by combining different self-supervised tasks. TRP-BERT [21] represented protein sequences by fusing two approaches based on the support vector machine classifier and contextual word embedding of BERT. Lu et al. [22] proposed the CPCProt model, which divided protein sequences into fixed-size segments and trained an autoregressor to distinguish subsequent segments of the same protein from random protein segments, which effectively extracted local and global features by maximizing the mutual information task.

After obtaining the representations of molecules and proteins separately, we considered that predicting the DTA by only concatenating the two representations directly and feeding them into the deep learning model cannot accurately discover the intrinsic relationship between them. Therefore, the attention mechanism [23] was introduced into the model to solve this problem, which is a complex cognitive function to make the more valuable parts of input data play an essential role in decision-making, thereby improving the efficiency and accuracy of model. With the application of deep learning in the field of drug discovery, attention mechanisms are also widely used and improved. Kurata et al. [24] optimized the attention mechanism architecture by exploring different depths of attention layer and context matrixes, which also demonstrated a plain attention mechanism can achieve high performance. Yang et al. [25] introduced multi-head attention and position-aware attention to the DTI prediction to improve the predictive and explanation ability of the model.

However, there are still several shortcomings of DTA prediction based on deep learning. First, the existing methods in the MPNN framework could not aggregate atom (node) or bond (edge) information effectively. Since bond information also plays an essential part in the graph, only focusing on the information of the atom leads to an incomplete representation of the molecular graph. Second, with the development of self-supervised learning, the representation of proteins using self-supervised learning could effectively utilize the existing large amount of protein sequence data. At the same time, due to the superiority of the BERT method in NLP, how to improve it to adapt to protein sequences is also a key issue to be addressed in this paper.

Based on this, a model combining undirected cross graph message passing neural network (undirected-CMPNN) for molecule and MLM with contrastive predictive coding to protein sequences (MCPCProt) for the target is proposed in this paper for DTA prediction. In addition, an attention mechanism is integrated into the model to discover the important relationship between drugs and targets by adjusting the weights.

## Methods

**Fig. 1:**
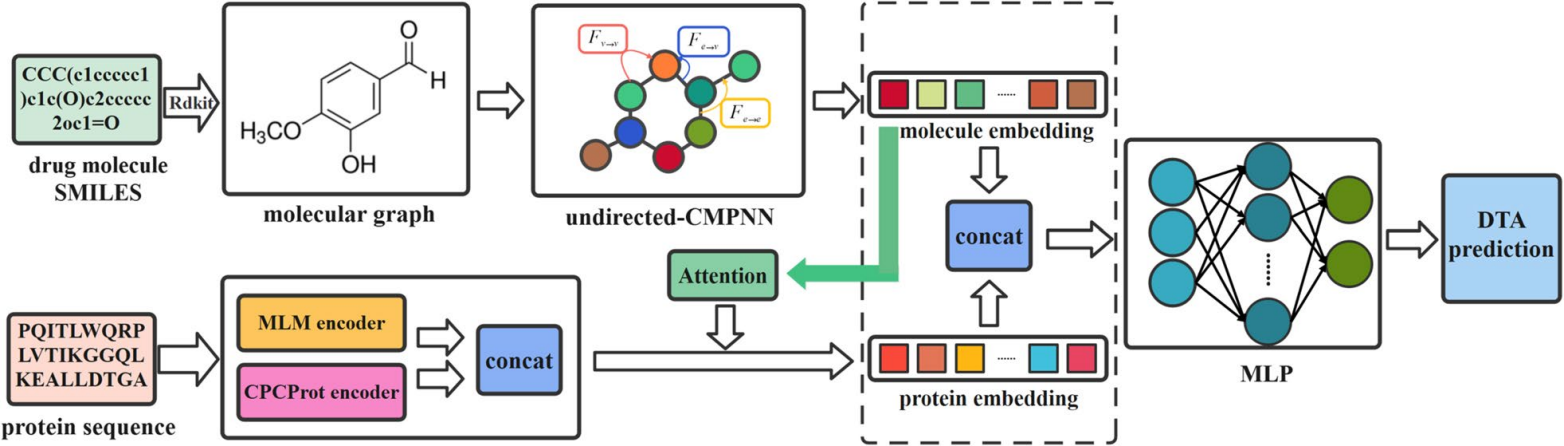
Model architecture

The proposed algorithm process is shown in Fig. 1. The input of the molecule is in SMILES format, which is converted to the graph structure, and the undirected-CMPNN is used to update the information of atoms and bonds in the molecular graph to obtain the representation of the entire molecule. The protein input is in the form of amino acid sequences. In this paper, inspired by CPCProt [22] and BERT [18], the MCPCProt self-supervised learning method is proposed, and 50,000 protein sequences were fed into the MCPCProt model for pre-training. The CPCProt and MLM embeddings of proteins are concatenated together as the representation of the protein. Considering whether the model can learn the important part of the protein for binding affinity, an attention mechanism is introduced. Finally, representations of the protein and molecule are concatenated and fed into the MLP to predict the binding affinity.

### Undirected-CMPNN for molecular representation

**Fig. 2:**
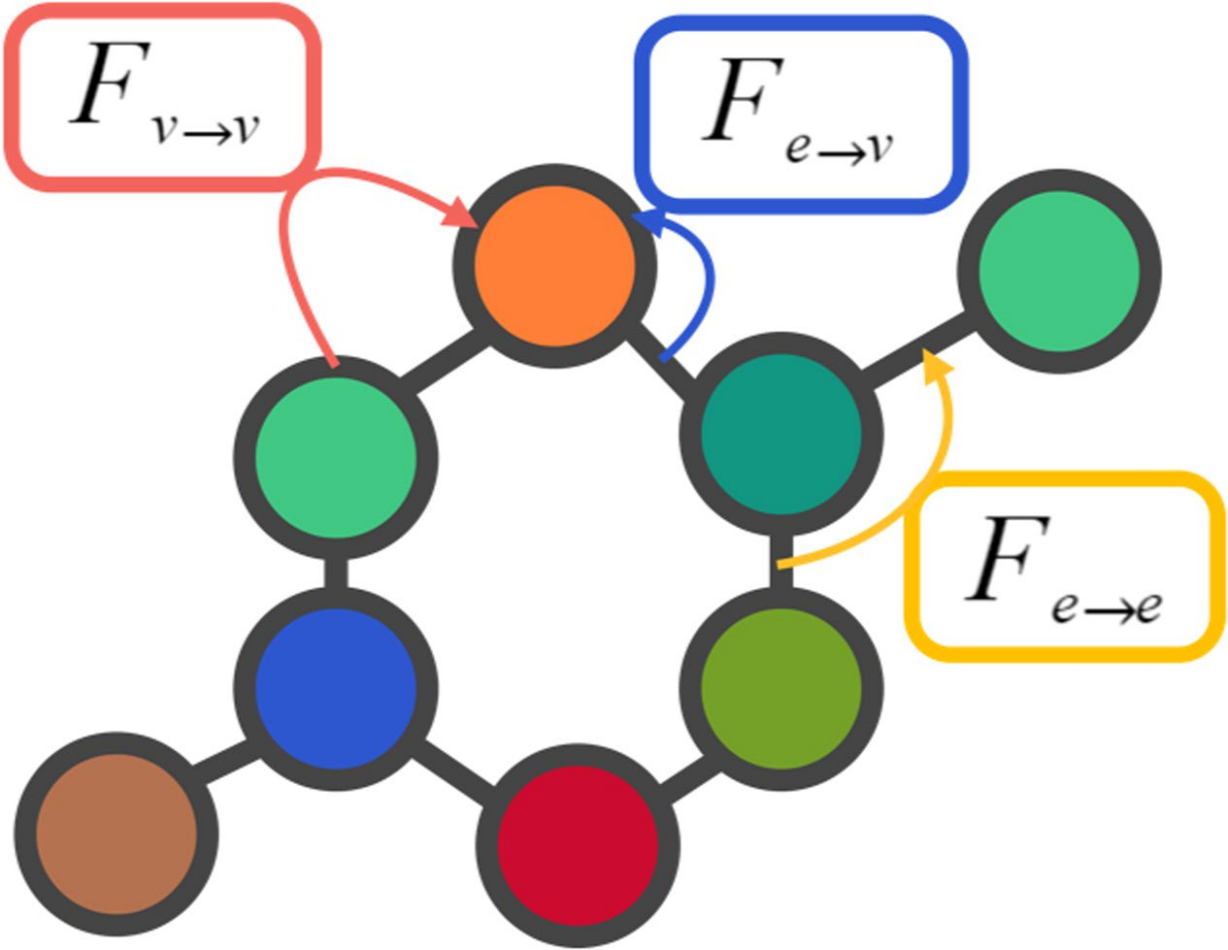
Messaging types of undirected-CMPNN

**Fig. 3:**
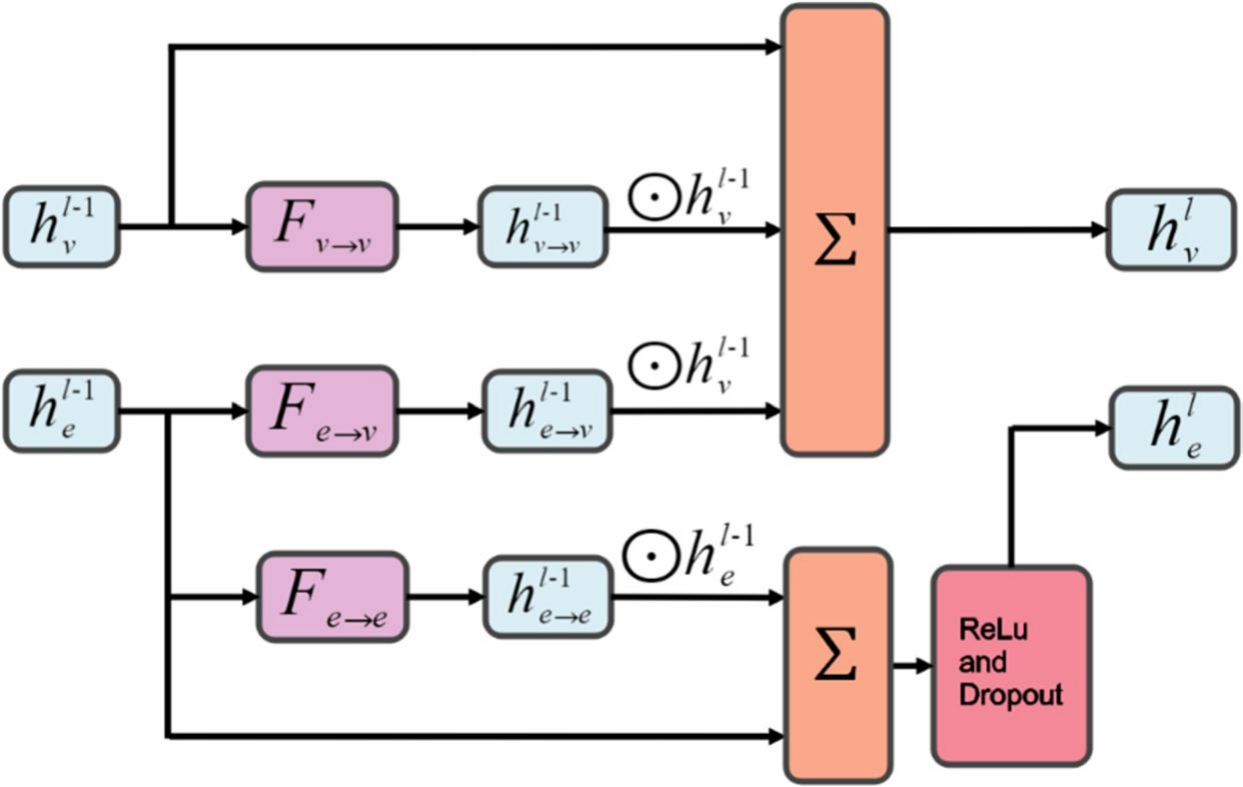
Message passing process of undirected-CMPNN

For the feature extraction of molecule, inspired by CMPNN [17], the undirected cross-messaging passing neural network (undirected-CMPNN) is proposed for molecular representation. There are three types of undirected-CMPNN message passing, as shown in Fig. 2, which are denoted as atom-to-atom $F_{v\rightarrow v}$, bond-to-bond $F_{e\rightarrow e}$, and bond-to-atom $F_{e\rightarrow v}$ message passing functions, respectively. The message-passing neural network algorithm is used to update the atomic and bond messages in the molecular graph, and the molecular representation is obtained by aggregating all atom features. These three messaging methods improve the generalizability of the model by enhancing the interaction of information between atoms and bonds. The advantage of undirected messaging method is that it allows for flexible interaction between atoms and bonds. An atom(bond) can directly pass a message to all atoms(bonds) adjacent to it without regard to the direction of the edges. When an atom(bond) receives a message, it can pass the message to its neighboring atoms(bonds), thus propagating the information throughout the graph. This information sharing process assists the model to compute and integrate both local and global information about the molecule. The detailed process of undirected-CMPNN is shown in Fig. 3.

The original bonds and atoms in the molecular graph are encoded into an initial hidden features $h_{e}^{0}$ and $h_{v}^{0}$ through Eqs. (1) and (2):

1 $$\begin{aligned} h_{e}^{0}= \sigma (Linear(x_{e}))) \end{aligned}$$

2 $$\begin{aligned} h_{v}^{0}= \sigma (Linear(x_{v}))) \end{aligned}$$

where $x_{e}$ and $x_{v}$ are the “one-hot” vector of bond and atom, *Linear* is the linear transformation, and $\sigma$ is the ReLU activation function. For the first layer, the atom features $h_{v}^{0}$ and bond features $h_{e}^{0}$ are fed into to the message passing neural network. For atom information updating, two methods are defined, including, the function $F_{e\rightarrow v}$ updating by neighboring bond, and the function $F_{v\rightarrow v}$ updating by neighboring atoms.

The function $F_{e\rightarrow v}$ is used to aggregate the hidden information of the adjacent incoming bonds to a specified atom *v*. It is similar to CMPNN, where the hidden state of the atom depends on the adjacent incoming bonds with the highest information intensity, so the maximum pooling method is adopted to update the atom information as shown in Eq. (3):

3 $$\begin{aligned} F_{e\rightarrow v}\left( h_{v}^{l-1} \right) = Maxpooling\left( \underset{e_{i}\in N_{e}\left( v \right) }{\underbrace{{h_{e_{i}}^{l-1}}{}}} \right) \bigodot \sum \limits _{e_{i}\in N_{e}\left( v \right) }\left( {h_{e_{i}}^{l-1}} \right) \end{aligned}$$

where $N_{e}(v)$ denotes the set of all adjacent incoming bonds connected to atom *v*. $\bigodot$ is the element-by-element multiplication of two features. The Eq. (4) ensures that the information of adjacent incoming bonds connected to atom *v* aggregate into $h_{e\rightarrow v}^{l-1}$.

4 $$\begin{aligned} h_{e\rightarrow v}^{l-1} = h_{v}^{l-1} \bigodot F_{e\rightarrow v}\left( h_{v}^{l-1} \right) \end{aligned}$$

In addition to $F_{e\rightarrow v}$, the function $F_{v\rightarrow v}$ have be introduced to the atom update method, which aggregates the hidden information of the adjacent atoms to a specified atom *v* as shown in Eq. (5). Then the atom aggregated representation $h_{v\rightarrow v}^{l-1}$ is computed by the update Eq. (6).

5 $$\begin{aligned} F_{v\rightarrow v}\left( h_{v}^{l-1} \right) = Maxpooling\left( \underset{v_{i}\in N_{v}\left( v \right) }{\underbrace{{h_{v_{i}}^{l-1}}{}}} \right) \bigodot \sum \limits _{v_{i}\in N_{v}\left( v \right) }\left( {h_{v_{i}}^{l-1}} \right) \end{aligned}$$

6 $$\begin{aligned} h_{v\rightarrow v}^{l-1} = h_{v}^{l-1} \bigodot F_{v\rightarrow v}\left( h_{v}^{l-1} \right) \end{aligned}$$

where $N_{v}(v)$ denotes the set of all atoms connected to atom *v*.

For bond features update, the neighboring bonds of the current bond, which share the same atom, are updated by the Eq. (8).

7 $$\begin{aligned} F_{e\rightarrow e}\left( h_{e}^{l-1} \right) = Maxpooling\left( \underset{e_{i}\in N_{e}\left( e \right) }{\underbrace{{h_{e_{i}}^{l-1}}{}}} \right) \bigodot \sum \limits _{e_{i}\in N_{e}\left( e \right) }\left( {h_{e_{i}}^{l-1}} \right) \end{aligned}$$

8 $$\begin{aligned} h_{e\rightarrow e}^{l-1} = h_{e}^{l-1} \bigodot F_{e\rightarrow e}\left( h_{e}^{l-1} \right) \end{aligned}$$

where $N_{e}(e)$ denotes the set of all neighboring bonds to specified bond *e*.

Then, the hidden features $h_{v\rightarrow v}^{l-1}$, $h_{e\rightarrow v}^{l-1}$ and $h_{v}^{l-1}$ are summed to generate the atom feature at layer *l* through Eq. (9), and the embedding of the bond was calculated through Eq. (10).

9 $$\begin{aligned} h_{v}^{l} = h_{e\rightarrow v}^{l-1}+ h_{v\rightarrow v}^{l-1}+h_{v}^{l-1} \end{aligned}$$

10 $$\begin{aligned} h_{e}^{l} = Dropout\left( \sigma \left( Wh_{e\rightarrow e}^{l-1}+h_{e}^{l-1} \right) \right) \end{aligned}$$

where $\sigma$ is the ReLU activation function and *W* is the learnable weight.

After *L* layers of undirected-CMPNN, the embedding of atom and bond are aggregated. Then, the $h_{v}^{L}$, $h_{v\rightarrow v}^{L}$, and $h_{e\rightarrow v}^{L}$ are concatenated and fed into the same communicative functions as the CMPNN to obtain the final embedding of the molecule.

Compared with CMPNN, the undirected-CMPNN is updated in two different ways. For bond information update, the bonds of the molecular graph are treated as undirected bonds by our method, thus bond information is updated from all neighbor bond features instead of removing its inverse bond features like CMPNN. For the atom messages update, the undirected-CMPNN updates the message from the neighbor atoms and adjacent incoming bond information, while CMPNN only uses adjacent incoming bond information for updating.

### MCPCProt for target feature extraction

To extract protein features more comprehensively, the MCPCProt is proposed in this paper, which combines the MLM method and the self-supervised feature extraction method of the CPCProt model. The MCPCProt could make full use of the bidirectional language learning of the MLM model and local and global information from CPCProt.

**Fig. 4:**
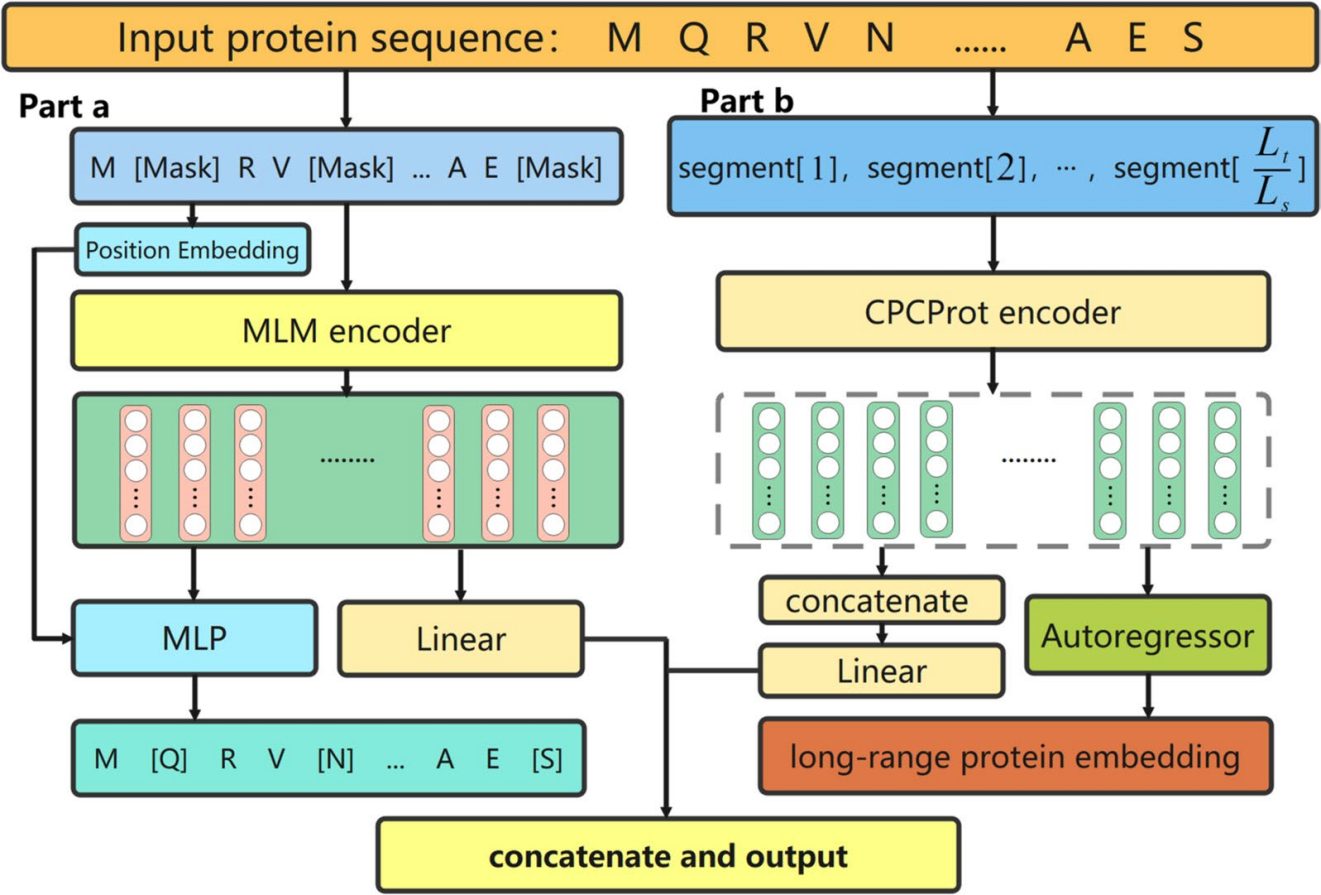
MCPCProt model

As shown in Fig. 4, the input to MCPCProt is the protein amino acid sequence, which is fed into two parts, including the MLM encoder and the CPCProt encoder respectively. We utilize different pre-training tasks to improve the comprehensiveness of protein representation. Each part of the MCPCProt is described below:

The task of MLM is to randomly mask 15$\%$ of the words in the protein sequence and then the contextual information is used to predict the masked words. As shown in the left part of Fig. 4, amino acids are randomly selected in the protein sequence for masking, and then the masked sentences are input to the MLM encoder for encoding. At the same time, a position embedding feature is generated combined with an output of MLM encoder, which is fed into Multilayer Perceptron (MLP) to output the predicted amino acids results and calculate the loss for optimization. MLM could effectively alleviate the problem of collecting contextual information. Through pre-training on a large-scale dataset, the embedding of the protein sequence is obtained as the input to the subsequent DTA prediction task.

In addition, the protein sequences could also be represented by the CPCProt self-supervised task as shown in the right part of Fig. 4. CPCProt divide the protein sequence into *x* segments and feeds them into the encoder separately, which includes multiple layers of convolution layer, normalization layer, and ReLU. The total protein sequence length is $L_{t}$ and each segment length is $L_{s}$, therefore x=$L_{t}/L_{s}$. The length of each segment needs to be determined based on the total length of the protein sequences, proteins that exceed the pre-defined length would be discarded, and proteins whose length is less than the pre-defined length would be padded. The output of each encoder is regarded as information covering each local part of the protein sequence. Moreover, the gated recurrent unit is used by the autoregressor to aggregate local information into a long-range protein embedding as a global feature. The autoregressor is optimized by using protein embeddings to distinguish between sequential segments of the same protein and random segments of the other proteins, which ensure that the CPCProt encoder contains both local and global information of protein sequence. For the DTA task, all of the local features are eventually concatenated as the protein feature.

### Attention mechanism

**Fig. 5:**
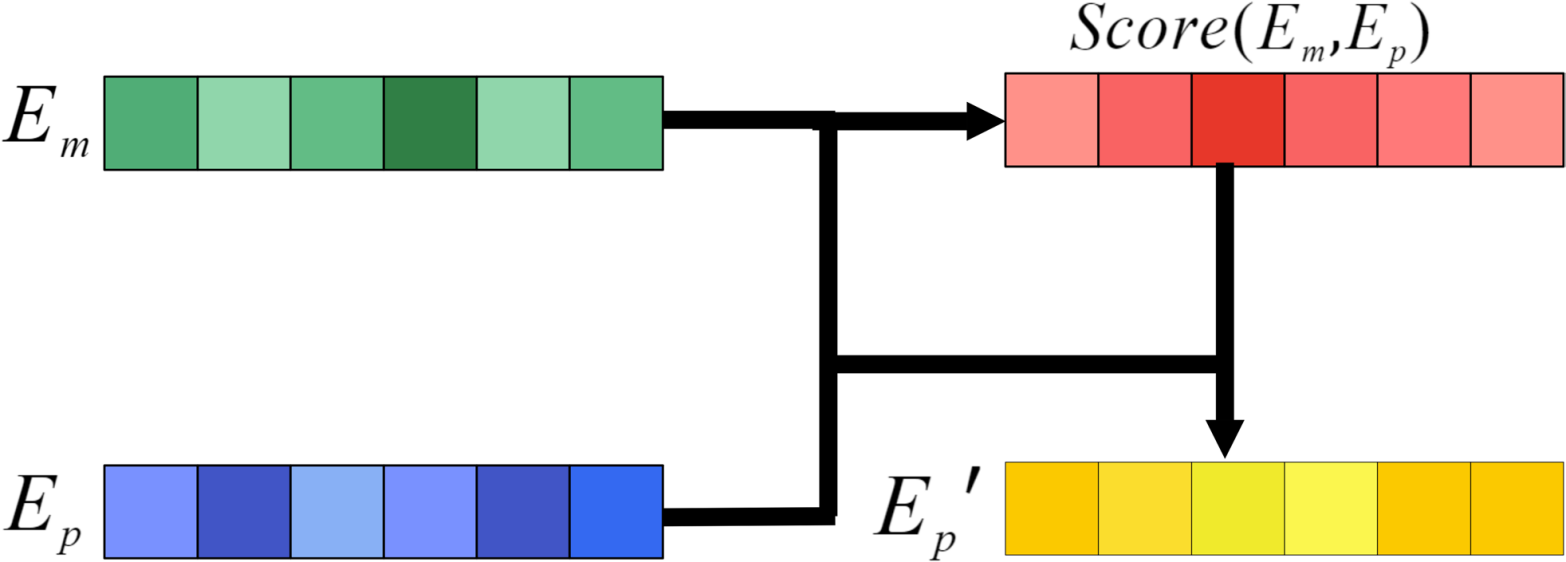
Attention mechanism

To explore the important part of protein sequences in the DTA prediction task, the attention mechanism in GanDTI [14] is applied in our model. As shown in Fig. 5, the attention mechanism achieves the goal of selecting the more critical information for the current task from a large amount of information. Specifically, the attention score is calculated based on the correlation between the input protein and molecular embeddings, and then the scores are used to re-weight the protein embeddings to obtain the final protein embeddings. The specific calculation process is as follows:

The *tanh* function is used to generate the attention score through the dot-product between the embedding of protein and molecule through Eq. (11):

11 $$\begin{aligned} Score(E_{m} ,E_{p})= tanh ( \sigma (W_{m} E_{m} )*(\sigma (W_{p} E_{p} ) )^{T} ) \end{aligned}$$

where $E_{m}$ and $E_{p}$ are the embeddings of molecule and protein and $\sigma$ is the *RELU* activation function. $W_{m}$ and $W_{p}$ are learnable weight matrices. The calculated scores can help the trained model focus on the important part of the protein sequence, and the embedding of protein is obtained through Eq. (12):

12 $$\begin{aligned} E_{p}^{'}= W_{s} Score(E_{m},E_{p})^{T}\sigma (W_{p}E_{p}) \end{aligned}$$

where $W_{s}$ is the learnable weights.

### MLP for DTA prediction

After obtaining the embeddings of molecule and protein, they are concatenated to generate features for MLP processing for DTA prediction as Eq. (13):

13 $$\begin{aligned} \hat{y} = MLP([E_{m},E_{p}^{'}]) \end{aligned}$$

where $\hat{y}$ denotes the predicted value, *n* denotes the number of samples and [ ] denotes the concatenation operation. The object of the model is to minimize the mean squared error, as defined in Eq. (14):

14 $$\begin{aligned} L_{MSE} = \frac{1}{n}\sum \limits _{i=1}^{n}(\hat{y}_{i}-y_{i} )^2 + \frac{\partial }{2}\left\| \Theta \right\| ^{2} \end{aligned}$$

where $y_{i}$ is the actual measured value, $\hat{y}_{i}$ is the predicted value, $\Theta$ is the combination of weights and bias in the network, and $\partial$ is the *L*2 regularization hyperparameter.

## Results

### Dataset

Ki dataset [14] and Davis dataset [26] are used to verify the effectiveness of our method. The values of Ki dataset were selected from the BindingDB dataset [27]. The Davis dataset is a well-known benchmark dataset that contains selectivity assays of the kinase protein family and the relevant inhibitors with their respective $K_{d}$ values. The detailed information about these two datasets is shown in Table 1. To verify generalization ability, our experiments also split the dataset into training, validation, and test sets in a ratio of 8:1:1. In addition, considering that self-supervised learning requires a large number of training samples, 50,000 protein sequence samples were selected randomly with lengths of $70-1000$ from the UniRef database [28] in the two pre-training tasks of the MCPCProt module.

**Table 1:** Information of the Ki dataset and the Davis dataset

Dataset	#Protein	#Drug	#Binding entries	Measured values type
Ki	112	2986	5000	$K_{i}$ (inhibition constant)
Davis	442	68	30056	$K_{d}$ (dissociation constant)

### Evaluation metric

#### Mean Squared Error (MSE)

MSE measures the average squared difference between the predicted values and the actual values, as shown in Eq. (15).

15 $$\begin{aligned} MSE = \frac{\sum _{i=1}^{n}(y_{i}-{\hat{y}}_{i})^{2}}{n} \end{aligned}$$

where *n* is the sample size, $y_{i}$ is the predicted value, and ${\hat{y}}_{i}$ is the actual value.

#### Concordance Index (CI)

CI measures whether the predicted binding affinity values in the same order as their true values, as shown in Eq. (16).

16 $$\begin{aligned} CI=\dfrac{1}{Z}\sum \limits _{\delta _{i} >\delta _{j}}h\left( b_{i}-b_{j}\right) \end{aligned}$$

where $b_{i}$ is the prediction value with the larger affinity $\delta _{i}$, $b_{j}$ is the prediction value with the smaller affinity $\delta _{j}$, *Z* is a normalization constant, and *h*(*x*) is the step function [29]. The step equation *h*(*x*) shown in Eq. (17).

17 $$\begin{aligned} h(x) = \left\{ \begin{array}{cc} 1, &{} \text {if } x>0\\ 0.5, &{} \text {if } x=0\\ 0, &{} \text {if } x<0 \end{array}\right. \end{aligned}$$

### Hyperparameters experiment

**Fig. 6:**
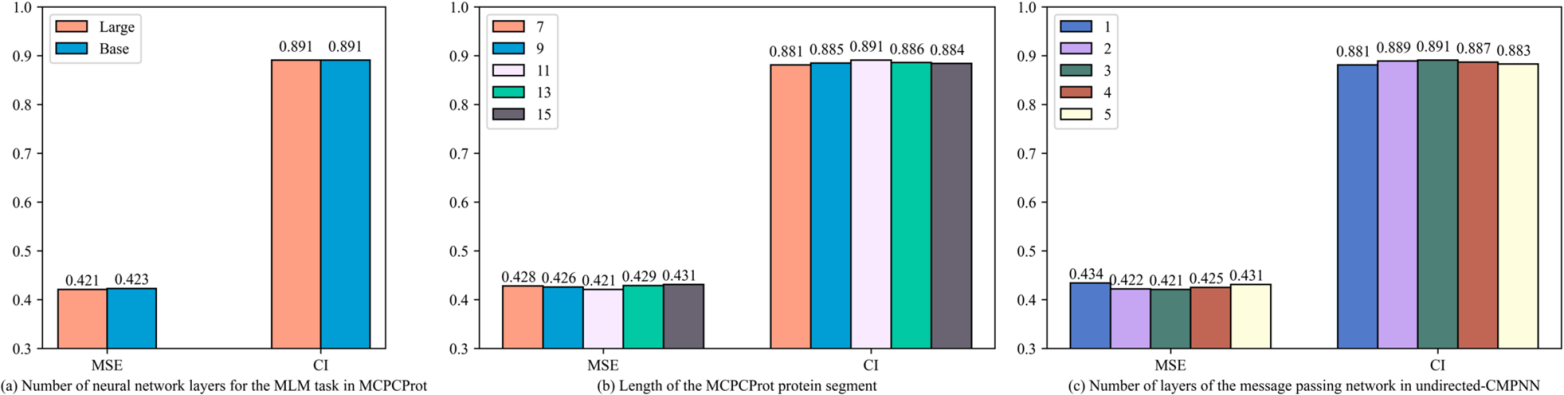
Hyperparametric experimental results

As shown in Fig. 6, to investigate the impacts of hyperparameters on the experimental results, we conducted experiments on the following hyperparameters using the Ki dataset: the number of neural network layers for the MLM task in MCPCProt: {Base, Large}, the length of the MCPCProt protein segment: {7, 9, 11, 13, 15} and the number of layers of the message passing network in undirected-CMPNN: {1, 2, 3, 4, 5}.

The differences between the Base and Large versions of the MLM task in MCPCProt are the number of layers of the neural network, the number of multi-headed attention, and the dimensionality of the hidden layers. In contrast, the Large version has adequate training parameters and more training steps, which ensure that the model is more accurate with a longer training time. As shown in Fig. 6 (a), there was no significant difference between these two versions, indicating that fewer parameters in the prediction task could also extract relatively accurate features.

The length of segments of proteins in MCPCProt determines the degree of detail of local and global information. The appropriate length is a crucial factor to ensure the performance of the model. A longer segment would lose certain local information, and a shorter segment would affect the aggregation process of global information. As shown in Fig. 6 (b), the best result was obtained when the length of the segment takes the value of 11, and this performance was consistent with the CPCProt experimental results. It is proved that the appropriate length of the segments is beneficial for MCPCProt to collect the local hidden features.

The depth of undirected-CMPNN, i.e., the depth of the neural network, reflects the range of atom and bond message passing. The greater the depth of the message passing, the farther the relative distance of the message passes. As shown in Fig. 6 (c), the best performance was achieved when the depth was 3 layers, and the performance of 2 layers was close to that of 3 layers. The smaller depth would lead to insufficient message interaction, and the larger depth would lead to over-smoothing of the molecular representation.

### Results of DTA prediction task

In this section, we presented the performance of our model in DTA prediction tasks. To demonstrate the superiority of the proposed method, we compared it with other previous models, including GanDTI [14], GraphDTA [9], WGNNDTA [13], and MGraphDTA [10], as shown in Table 2. The results of these methods were acquired by training the model using the source code provided by them. That is to say, we used the same datasets and dataset partition method to ensure the effectiveness of the comparative experiment. The MSE and CI values of the proposed method were outperformed other methods. GanDTI, as they mentioned in their paper, uses a simple model for the prediction task of DTA, however, both proteins and molecules contain a great deal of information, thus it is difficult to guarantee the accuracy just by using a simple model. In GraphDTA and MGraphDTA, the graph neural network ignores the message interactions of atoms and bonds, which will not embed more accurate information into the molecular features. For protein embedding, only multilayer CNNs are used, which is not enough to extract more valuable information about the protein sequence. In WGNNDTA, the GNN used to extracting molecular feature also only takes into account the information of the atoms in the graph, in addition, the constructed weighted protein graph may introduce new errors during the construction process and affect the performance of DTA. Our model could predict binding affinity with high accuracy which mainly depended on two major advantages of the model. First, the undirected-CMPNN fully takes into account the message passing of atoms and bonds in the molecular graph, which improves the accuracy of molecular features. Second, the combination of the MLM and CPCProt model could extract local and global information, which also improves the robustness of the protein feature. It is worth mentioning that the introduction of the attention mechanism also improves the performance of the model.

**Table 2:** Comparison of DTA prediction performance on Ki and Davis dataset

Model	MSE (Ki)	CI (Ki)	MSE (Davis)	CI (Davis)
GanDTI	0.469	0.878	0.236	0.885
GraphDTA	0.441	0.881	0.225	0.895
WGNNDTA	0.430	0.886	0.211	0.898
MGraphDTA	0.427	0.889	0.205	0.899
Our model	0.421	0.891	0.203	0.900

### Ablation study

To further analyze the impact of different factors on the model, the ablation experiments were implemented on Ki dataset, and all parameters were consistent except the one to be evaluated.

**Fig. 7:**
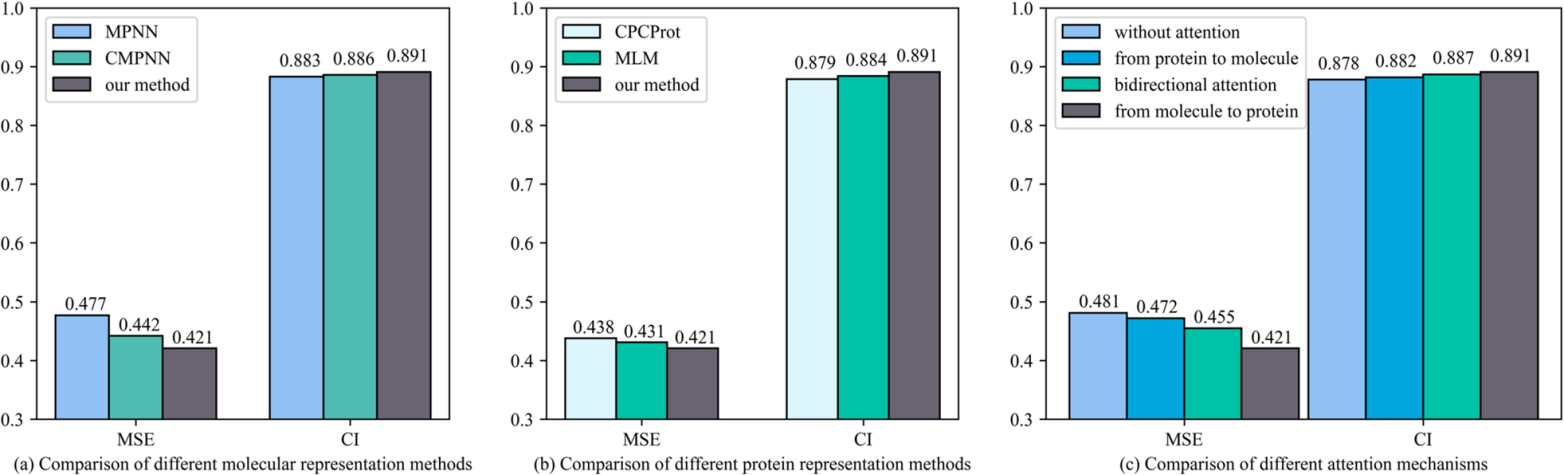
Comparison of different molecular representation methods

In this paper, an undirected-CMPNN model is proposed to extract molecular features. In order to demonstrate the advantage of the undirected-CMPNN model, we compared our method with other different molecular representations methods including the MPNN and CMPNN, the experimental results are shown in Fig. 7 (a). The CMPNN model could pay more attention to the atom-bond messaging compared with the traditional MPNN. Moreover, the proposed undirected-CMPNN model could consider more comprehensive atom and bond messages compared with the traditional CMPNN, and the performance was further improved, with MSE 0.421 and CI 0.891, which was higher than the other two.

In addition, the protein embedding method combining MLM and CPCProt is proposed in this paper. To demonstrate the effectiveness of this combination method, we also compared the MCPCProt with CPCPort. Both MSE and CI coefficients were improved after adding the MLM on CPCProt, which enables the protein sequence to better learn the contextual information, the experimental results are shown in Fig. 7 (b).

The attention mechanism is utilized to capture the important part of protein for the DTA task. In this section, we tested and compared our attention mechanism (from molecule to protein) with the other three methods including, bidirectional attention, and attention from protein to molecule, and without attention, the results are shown in Fig. 7 (c). The attention from molecule to protein performed better than other attention mechanisms. The method with attention from protein to molecule had not achieved higher performance. It is possible that the number of proteins is less than the number of molecules, which leads to attention mechanism appearing more redundant information. Moreover, the performance of bidirectional attention was not as good as that of attention from molecule to protein.

### Alpha-2A adrenergic receptor for case study

To demonstrate the effectiveness of our method, the case study was conducted using drug-target pairs that have been verified as bindable pairs from DrugBank [30], following the approach in MSF-DTA [31]. Specifically, pre-trained model weights were loaded into our model to evaluate the binding intensity of the Alpha-2A adrenergic receptor and 1665 drugs. Then, we ranked 17 known drugs that interact with this protein in descending order based on predicted affinity. The results are shown in Table 3. From the table, we can conclude that all 17 drugs are within the top 40%, with 9 drugs ranking in the top 9% and 13 drugs in the top 15%. It is demonstrated that our research is effective in real-world scenarios and has great potential for practical applications. It is worth noting that, to ensure the validity of the case study, the Alpha-2A adrenergic receptor was not included in our training dataset.

**Table 3:** Drug ranking with the Alpha-2A adrenergic receptor

Drug	Ranking	Drug	Ranking	Drug	Ranking	Drug	Ranking
DB00633	22	DB00449	86	DB01018	196	DB00692	535
DB06262	23	DB11273	92	DB01392	215	DB13345	660
DB06707	51	DB00368	98	DB00968	246		
DB04855	62	DB01608	139	DB11278	252		
DB00852	63	DB00668	192	DB00193	437		

## Discussion

In this paper, we used undirected-CMPNN to represent molecules and MCPCProt to represent proteins. Compared with CMPNN, the undirected-CMPNN is updated in two different ways to represent molecules, which could consider and cover the information from atoms and bonds in the molecular graph. The proposed undirected-CMPNN could improve the accuracy of DTA prediction, which was demonstrated in the experiment part. Moreover, the MCPCProt model combining the MLM and CPCProt model also improves the robustness of the protein representation, since it can extract local and global information.

In order to ensure the model could focus on the important part of the protein, the attention mechanism is introduced in the model, which also improves the performance of the model. It is worth mentioning that we found that the bidirectional attention mechanism does not work better than attention from molecule to protein, which is different from the results of other work, and we believe that it is caused by the imbalance between the amount of data of proteins and molecules.

However, the interpretability of deep learning has always been a challenge. Although some intuitive explanations have been provided by attention mechanisms in certain studies, the attention mechanism used in this paper has delved deep into the feature level of proteins. At the feature level, the importance of different dimensions can be explored, but explanations at the data level cannot be provided. In future work, the interpretability problem will also be further investigated and studied.

## Conclusion

The task of DTA prediction is important to drug discovery and drug screening. Deep learning is helpful and effective for this task without requiring highly specialized biological knowledge, which reduces the cost of research. In this paper, we propose a DTA prediction model using an undirected-CMPNN for molecule embedding and MCPCProt models for protein embedding. Both embeddings are concatenated for DTA prediction. The results showed that the proposed model outperformed other deep learning methods, which also provides a novel strategy for deep learning-based virtual screening methods.

## Data Availability

The code and data are provided at https://github.com/XiaLeiming/UCMCDTA.

## Abbreviations

DTA: Drug-target affinity
DL: Deep learning
SMILES: Simplified molecular input line entry system
RNN: Recurrent neural network
LSTM: Long short-term memory network
DTI: Drug-target interaction
GNN: Graph neural network
GCN: Graph convolutional neural networks
CNN: Convolutional neural network
MPNN: Message passing neural network
NLP: Natural language processing
BERT: Bidirectional encoder representations from transformers
MLM: Mask language model
MCPCProt: MLM with contrastive predictive coding to protein sequences
Undirected-CMPNN: Undirected cross graph message passing neural network
MLP: Multilayer perceptron
Ki: Inhibition constant
MSE: Mean squared error
CI: Concordance Index
